# Partial Attention in Global Context and Local Interaction for Addressing Noisy Labels and Weighted Redundancies on Medical Images

**DOI:** 10.3390/s25010163

**Published:** 2024-12-30

**Authors:** Minh Tai Pham Nguyen, Minh Khue Phan Tran, Tadashi Nakano, Thi Hong Tran, Quoc Duy Nam Nguyen

**Affiliations:** 1 Faculty of Advanced Program, Ho Chi Minh City Open University, Ho Chi Minh City 700000, Vietnam; 2151013086tai@ou.edu.vn; 2 Faculty of Information Technology, Ho Chi Minh City Open University, Ho Chi Minh City 700000, Vietnam; 3 Department of Core Informatics, Graduate School of Informatics, Osaka Metropolitan University, Osaka 558-8585, Japan

**Keywords:** attention mechanism, deep learning, noisy labels, weighted redundancies

## Abstract

Recently, the application of deep neural networks to detect anomalies on medical images has been facing the appearance of noisy labels, including overlapping objects and similar classes. Therefore, this study aims to address this challenge by proposing a unique attention module that can assist deep neural networks in focusing on important object features in noisy medical image conditions. This module integrates global context modeling to create long-range dependencies and local interactions to enable channel attention ability by using 1D convolution that not only performs well with noisy labels but also consumes significantly less resources without any dimensionality reduction. The module is then named Global Context and Local Interaction (GCLI). We have further experimented and proposed a partial attention strategy for the proposed GCLI module, aiming to efficiently reduce weighted redundancies. This strategy utilizes a subset of channels for GCLI to produce attention weights instead of considering every single channel. As a result, this strategy can greatly reduce the risk of introducing weighted redundancies caused by modeling global context. For classification, our proposed method is able to assist ResNet34 in achieving up to 82.5% accuracy on the Chaoyang test set, which is the highest figure among the other SOTA attention modules without using any processing filter to reduce the effect of noisy labels. For object detection, the GCLI is able to boost the capability of YOLOv8 up to 52.1% mAP50 on the GRAZPEDWRI-DX test set, demonstrating the highest performance among other attention modules and ranking second in the mAP50 metric on the VinDR-CXR test set. In terms of model complexity, our proposed GCLI module can consume fewer extra parameters up to 225 times and has inference speed faster than 30% compared to the other attention modules.

## 1. Introduction

**Figure 1:**
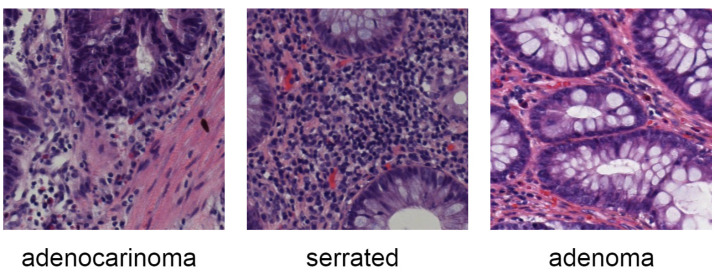
Several samples of noisy labels in medical imaging. Highly similar contexts can be observed among images, causing misleading model detections.

Medical imaging is one of the initial stages of examination to identify potential health risks. Recently, the increase in the number of new cases of several diseases [1] has been seriously burdening this first line of diagnoses. Therefore, the application of anomaly automatic detection on medical images has been urgent. Some initial research work was carried out as a first step of applying deep learning (DL) to build a computer-aided diagnosis (CAD) system [2,3]. However, there are various types of medical images, such as X-rays, Magnetic Resonance Imaging (MRI), Computed Tomography (CT), or even colon slides. Each type of image has its own features, and most of them contain a significant amount of noisy labels, including overlapping objects or the appearance of disease signals, which indicate similarities with its surrounding area like color and shape, which are discussed in many studies [3,4,5]. Figure 1 illustrates several samples of noisy labels on colon slides in the Chaoyang dataset [6], where the images belong to different classes but appear in the same patterns. Moreover, some serious rare diseases have only a few reported cases, which makes the number of images in the dataset used to train deep learning (DL) models challenging. So, these problems significantly hinder the convergence of the DL models and lead to inaccurate predictions. Hence, it is critical to address these matters in order to improve the performance of DL models. Inspired by the success of Transformers in NLP [7], the attention mechanism was proposed. This type of mechanism aims to imitate human vision concepts, which prioritize focusing on important features of objects rather than considering them all simultaneously. Currently, there are two noticeable types of attention mechanisms: the self-attention [7] and the soft attention [8]. By creating attention weights based on long-range dependencies, self-attention enables the model to capture important features and suppress non-related information, thereby constructing more valuable information and effectively providing visual features that can be beneficial in addressing noisy labels of medical imaging. Several works [9,10] indicate the potential of using self-attention to enhance the model’s performance under conditions of medical images. However, the mechanism is known for its quadratic complexity [11] that would increase computation cost, which ends up with extremely high memory consumption during training and inference. Moreover, self-attention would require a large amount of data for training to assist the convergence of the model; it tends to be less effective on small datasets or noisy labels, as indicated in previous work [12], and most of the deep learning models that enable the ability of self-attention tend to construct the whole architecture from scratch [9,13,14], which turns out to be a hindrance in architecture adaptation. Unlike self-attention, the soft attention mechanism aims to capture important features at a significantly lower resource consumption cost. Therefore, it is usually built as an attention module that can be integrated into any available DL models, enhancing the architecture’s flexibility.

Due to the aforementioned problems, we propose the integration of global context modeling, primarily designed to replicate the non-local interaction of self-attention and local interaction, which mainly capture channels by using 1D convolution. This approach significantly improves the visual features, enhancing their resilience to noisy labels while minimizing the complexity trade-offs. However, our initial attempt to combine local interaction with global context modeling was unsuccessful as it relied solely on a single branch to generate weights directly from the output of the global context modeling module. Therefore, after several conducted experiments, the study managed to propose the combination of two separated branches to create attention weights, including enhanced global context branch and conventional branch, which has shown its desired performance. After highlighting the characteristic features from these two branches, they are aggregated and passed through a 1D convolution that is capable of selecting important channels adaptively before being used for generating weights. This modification allows the module to gain both the ability to introduce robust attention weights and effectively address noisy labels on medical images. The new module can still model contexts like GC-net [15], but it uses a lot less memory and does not add as many extra parameters because it relies on local interactions instead of a bottleneck structure, which primarily uses 2D convolution for capturing. This module is then named the Global Context and Local Interaction (GCLI) module.

**Figure 2:**
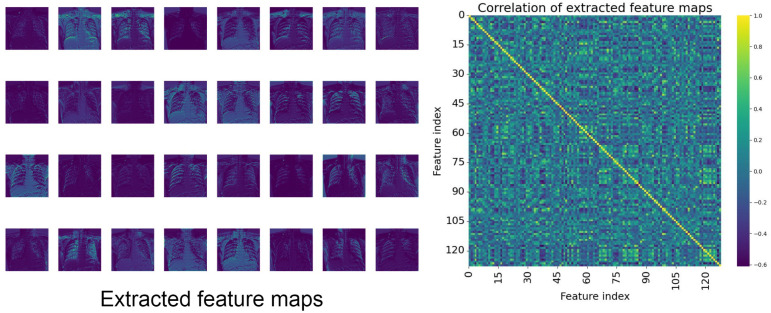
Extracted feature maps in the early layers of the DL model. Highly redundant features with strong correlations can be observed across channels. This potentially limits the model ability.

Redundancies are extracted features but do not provide any benefits during the classification phase due to their resemblance with other feature maps in complex DL models, as indicated in Figure 2. In fact, they may even deteriorate the feature maps and negatively impact the performance of deep learning models. Although the appearance of redundancies in many DL models has been stated in previous work [16], it is ambiguous if the attention mechanism, especially the combination of local interaction and global context, also creates irrelevant attention weights or weighted redundancies. There is the earlier work [17] that indicated the appearance of attention redundancies in many Transformer architectures. However, this research primarily focused on natural language processing (NLP) tasks, neglecting other computer vision tasks or the redundancies in attention mechanisms such as global context modeling. Hence, acknowledging the redundancies in the deep layers of DL models, another question arises: “Will our attention module introduce the redundant weighted features to the network?”. Therefore, to answer this question, we introduce the partial attention strategy to the GCLI module, which utilizes a half-number of input channels for the attention weight generation rather than considering all channels. By applying the proposed strategy, the experiments indicate that the modified GCLI module, which uses only half the number of channels, has moderately higher performance compared to the original GCLI module. This result shows that the original GCLI module tends to introduce weighted redundancies, and the proposed partial attention strategy can be effective at addressing this problem. Also, it indicates that attention modules may have the potential risk of introducing even redundant weights to the DL models. Eventually, both GCLI modules demonstrate a highly promising performance, surpassing most current SOTA attention modules in terms of both performance and resource consumption, thereby aiding the DL models in detecting anomalies on medical images. To summarize, our contributions can be described as follows:

- First, our study has succeeded in creating an attention module by combining local interaction and global context modeling that is able to perform the ability of long-range dependencies in self-attention with an extremely low complexity cost. This approach is effective in addressing the problem of noisy labels on many medical datasets.
- Second, our study has discovered the redundant weighted features created by modeling global context under the effect of noisy labels on medical images.
- Finally, our study has proposed a solution to tackle the issue of weighted redundancies called partial attention strategy, which involves using only half the number of channels to generate attention weights.

## 2. Related Works

For a decade, several approaches based on convolutional neural networks (CNNs) [18,19,20] were proposed for computer vision tasks on medical images. These works primarily utilized transfer learning and self-supervised learning, which mainly relied on the baseline architecture that may not be best suited when handling noisy labels on medical images. Therefore, further modern approaches from CNNs that aimed to perform similar abilities of self-attention in Transformers were introduced, such as ConvUNeXt [21], which was inspired by ConvNext [22]. This unique neural network aimed to reduce the parameters while achieving higher performance and was mainly designed for segmentation tasks. Another work [23] introduced the application of EfficientNet [24] to automatically diagnose COVID-19 signs on CT medical images. However, all these methods still did not address the noises in medical images effectively, as was the drawback of CNNs, which only capture local pixels determined by kernel size instead of considering other long-range spatial features. Moreover, CNNs had translation equivariance, which turned out to be a problem of many classification tasks in the small and noisy datasets.

In order to make the architectures more robust to noisy labels, attention mechanism approaches were proposed [25,26,27,28]. These studies introduced many attention modules such as Coordinate Attention (CA) [29], simple attention (simAM) [30], Global Context (GC), Convolution Block Attention Module (CBAM) [31], Efficient Channel Attention (ECA) [32], and Squeeze and Excitation (SE) [33], to assist models in determining where to focus. Nonetheless, the strength of these attention mechanisms was mainly creating attention weights for spatial features or channel information only. Yet, by creating weights for specific information, these previous modules were lacking the ability to suppress the existence of noisy labels due to weak visual features. Therefore, more works on advanced approaches such as Transformer-based networks were introduced [9,10,34,35,36]. As mentioned, the key to these proposed Transformers was self-attention, which constructed weights by adding the interaction of non-local features, but it was struggling with higher complexity. Other approaches tended to imitate the mechanism of self-attention with a lower complexity [15,37]; these approaches looked for a combination of pairwise relations and all positions to generate weights for specific query positions of features, resulting in making the process much simpler compared to the original approach of self-attention. However, the effect of non-local interaction in neural networks would only consider the global information for producing weights. Hence, during channel transformation, it skipped many potential local features to construct proper weights for addressing noises. This problem turned out to greatly reduce the mechanism ability in many small or noisy datasets.

The redundant features were proved to exist in many architectures with various topics in classification, object detection, and segmentation [38]. Hence, several works [39,40,41] aimed to exploit and propose different approaches to address the redundancies in convolution by reducing the parameters, but resulted in higher memory consumption due to increased model width in order to compensate for the decrease in accuracy. A further approach from the work of Francois Chollet [42] introduced the depthwise separable convolution that processed the convolution in a more efficient way and can reduce the effect of redundant features by applying a single kernel per input channel rather than convolving input channel with every kernel. Another approach [43] proposed the partial convolution, which took only one fourth of the input channel for convolution. This unique approach tended to perform better than depthwise separable convolution in many computer vision tasks in both performance and memory consumption. However, the effect of the partial convolution in the attention mechanism, especially with global context modeling, still remained undiscovered.

Because of these problems, our approach was proposed to use global context modeling combining with local interaction via 1D convolution to enhance the visual features by efficiently producing attention weights that were resilient to noisy datasets. Moreover, our work also introduced the mechanism similar to partial convolution for the attention mechanism to suppress redundant weighted features and unnecessary high computation from the previous non-local interaction approach.

## 3. Methodology

### 3.1. Local Interaction in Channel Transformation of Global Context Modeling

Global context modeling was first introduced in the work [15], aiming to replicate the long-range dependencies capturing of self-attention. The authors of the GC block proposed a bottleneck structure for channel transformation, which utilized the output of global context modeling as its input. This structure is similar to the one used in the SE-Net [33]. However, the bottleneck structure used 2D convolution to make attention weights, which was shown to be slow and hard to converge in earlier research [44], and our further analysis in this study also indicates the improper weight generation of this structure for addressing noisy labels. This drawback significantly restricts the ability of global context modeling in the GC block. Therefore, it is crucial to explore alternative solutions to effectively handle channel transformation, thereby enhancing the reliability and efficiency of the global context modeling module. To tackle this issue, our study examined and suggested substituting the bottleneck structure with local interaction. This local interaction method was first introduced in the work [32], where the authors proposed an efficient way to produce attention weights using 1D convolution instead of traditional 2D convolution methods. This local interaction allowed the model to learn attention features without any dimensionality reductions. To begin with our approach, let us first review the Equation (1) that indicates the process of the GC block.

(1) $$z_{i}=W_{v2}ReLU\left(LN\left(W_{v1}\left(X_{gc}\right)\right)\right)$$

In Equation (1), $W_{v1}$ is the first 2D convolution that shrinks the number of input channels, LN is the layer normalization, ReLU is the activation function ReLU, and $W_{v2}$ is the last 2D convolution used to increase the number of channels. The whole process indicates the result of channel transformation $z_{i}$ of the global context module’s output $X_{gc}$, where

(2) $$\begin{array}{c} X_{gc}=\Sigma_{j=1}^{N_{p}}\frac{\exp^{W_{k}x_{j}}}{\Sigma_{m=1}^{N_{p}}\exp^{W_{k}x_{m}}} \end{array}$$

**Figure 3:**
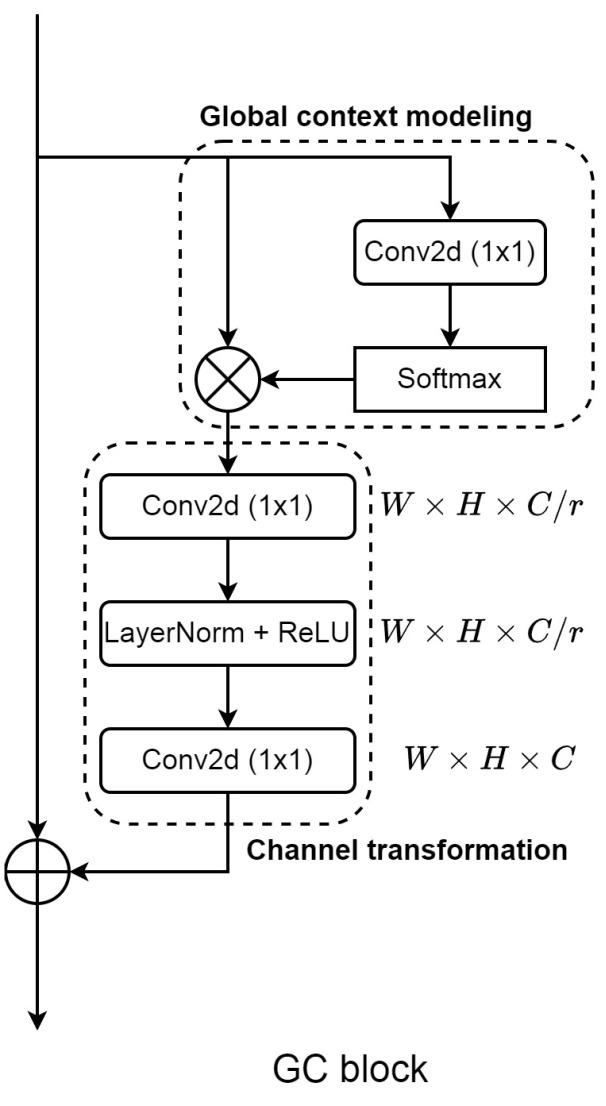
The structure of the GC block. This structure primarily integrates global context modeling with a bottleneck structure, which is used to generate attention weights. The bottleneck structure significantly restricts the capacity of modeling global contexts during channel transformation.

In Equation (2), *x* is the input channel, $N_{p}$ indicates the position in the feature map, and $W_{k}$ illustrates the matrices of linear transformation. This process indicates the creation of global attention weights by modeling the global context of featured maps. This equation computes the spatial information across spatial domains of every key position $N_{p}$ from input *x* in order to form the global context vector. This vector shares similar features with the long-range dependencies in self-attention. Both Equations (1) and (2) illustrate the process of the GC block. The structure of the GC block is illustrated in Figure 3 where Equation (1) is the global context modeling part and Equation (2) is the channel transformation part.

In terms of channel transformation or channel attention of the GC block, Equation (1) utilizes the bottleneck as the main component for creating channel weights. The structure itself normally adds difficulty during the model training phase [44] due to the effect of information loss caused by dimensionality reduction, which significantly reduces the ability of the original GC block, which primarily relied on the bottleneck structure to reweight the feature maps created by modeling global context vectors. This drawback may remarkably reduce the effect of addressing noisy labels of the module. Furthermore, the operation of 2D convolution in the bottleneck structure typically requires a significant amount of computation, which slows down the inference speed and convergence of DL models. Furthermore, the operation of 2D convolution in the bottleneck structure typically requires a significant amount of computation, which slows down the inference speed and convergence of DL models. Even though the previous work already addressed the problem by proposing the layer normalization, our experiments in the study indicate that the earlier approach did not achieve the desired output under the noisy labels on medical images. Instead, we adopt the idea of local interaction from the work [32] as the replacement for the bottleneck structure. When compared to SE-Net from the work [33], local interaction mostly builds weights through the interaction between channels, which is picked up by global average pooling and models the weighted channels with 1D convolution. This method not only avoids the problem of dimensionality reduction, keeping more useful information for attention weight generation, but it also uses fewer resources than the bottleneck structure.

Hence, Equation (2), which indicates the process of modeling global context, is adopted and further constructed with local interaction for channel transformation. However, the local interaction that was introduced from the previous work [32] is not just being applied conventionally. Instead, through the ablation analysis, with our observations of how the global context modeling phase works, we managed to find that applying the original way of local interactions on the global context feature level does not effectively capture weighted information as expected. Hence, to overcome the problem, global max pooling is applied to replace the global average pooling in the original local interaction; this unique pooling considers the most highlighted features among channels to represent the channel characteristics instead of global average pooling, which only captures the average features among channels. This proposal will enable the model to capture information in a robust and resilient manner even in the presence of unstable features. However, in the past, many works [45,46] proved the effectiveness of different global information descriptors, like global max pooling for retaining the most significant information or global average pooling normally to suppress the effect of irrelevant information. Moreover, another work [31] also indicates the combination of these two different pooling types; the authors stated that each type of pooling has its own unique way to capture the information to strengthen the attention weights. Since the goal is to obtain as much useful information as possible, using a single global pool may limit the proposed module’s usefulness. This is because the solution needs to provide unique attention weights that can both keep the most important information and assist the convergence of DL models under noisy label conditions. As a result, another branch of non-weighted or conventional features is passed to the global average pooling in order to provide more information. In detail, this branch acts as the noise-resilient descriptor, while the other branch, which passes the output of the global context module to the global max pooling, captures the most prominent features among channels. The process of two separate branches can be described as follows:

(3) $$F_{gmp}=GMP\left(X_{gc}\right)=max_{i=0}^{H-1}max_{j=0}^{W-1}X_{gc_{\left(i,j\right)}}$$

(4) $$F_{gap}=GAP\left(X_{n}\right)=\frac{1}{HW}\Sigma_{i=0}^{H-1}\Sigma_{j=0}^{W-1}X_{n_{\left(i,j\right)}}$$

According to Equation (3), GMP stands for global max pooling and $X_{gc}$ stands for global context weighted features. This pooling takes an input of size W×H×C, where *W* is the width, *H* is the height, and *C* is the number of channels, and gives back a list of characteristic features $F_{gmp}$ that is 1×1×C. The pooling takes the highest value in each feature map as the characteristic feature along channels. In Equation (4), GAP stands for global average pooling and $X_{n}$ stands for the non-weighted or conventional features. The pooling takes in $X_{n}$, which is the same size as $X_{gc}$, and gives back a list of characteristic features $F_{gap}$ that is 1×1×C. However, instead of using the highest value, this pooling computes the average value of each feature map to form the sequence.

At this point, based on the results of two different feature descriptors in Equations (3) and (4), it is essential that some features captured by global max pooling are more useful than those captured by global average pooling and vice versa. Therefore, in order to use the information captured by these poolings effectively, they are aggregated to form the combined characteristic sequence with size 2×1×C and passed through a 1D convolution with a large kernel size of 7×7 for the convolution to adaptively determine characteristic features that will be used to produce attention weights. The output of this step is the sequence of selected features with size 1×1×C. Since 1D convolution on an input size of 2×1×C is low on complexity cost, this process is now able to use a large kernel size to create a larger interaction between noisy label resilient features and strong features to obtain the proper weights. Moreover, in the GC block, the authors overcame the difficulty of the bottleneck structure in training by adding layer normalization between two convolutional layers. Our proposal is different, as we use an activation function SiLU [47] to further map the selected features to the more complex spaces, creating additional abstract features before being used as inputs for attention weight generation. As our approach already included the process of selecting potential features during the capturing information phase, the generated abstract features are able to reduce the risk of irrelevant features while providing better visual features compared to previous approaches. This whole process is indicated as follows:

(5) $$F_{s}=SiLU\left(\Sigma_{j=1}^{7\times 7}W^{j}Y_{s}^{j}\right),\forall Y_{s}^{j}\in {\left[F_{gmp}:F_{gap}\right]}^{2\times 1\times C}$$

(6) $$F_{att}=\Sigma_{j=1}^{k}W^{j}Y_{att}^{j},\forall Y_{att}^{j}\in F_{s}^{1\times 1\times C}$$

Equation (5) indicates the process of selecting prominent features from the aggregated channels based on two different types of pooling. Here, $\left[F_{gmp}:F_{gap}\right]$ is the aggregated sequence channel with size 2×1×C, $W^{j}Y_{s}^{j}$ is the 1D convolution operation on feature *Y* of channel *j* with kernel *W* having size 7×7, and SiLU is the activation function. The output of this process is the selected sequence channel $F_{s}$ size 1×1×C.

From the result of Equation (5), $F_{s}$ is further passed to Equation (6) to form the local interactions from selected sequence channels. The 1D convolution operation $W^{j}Y_{att}^{j}$ is used on the input $F_{s}$, which is 1×1×C. The kernel size *W* in this process is 3. This number was proved to be effective to form local interactions in the previous study of the original local interactions [32]. The output of this process is the attention weight $F_{att}$ with size 1×1×C. The final weighted channels are generated by passing $F_{att}$ to a sigmoid function and then multiplied with the original inputs.

**Figure 4:**
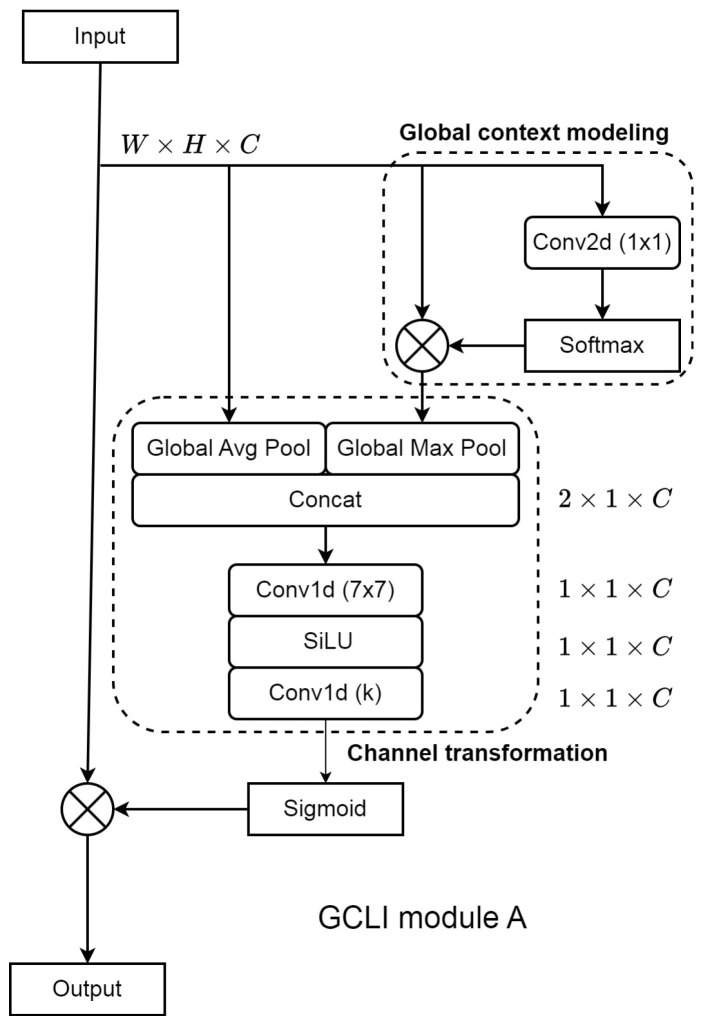
The structure of GCLI module A. This proposed structure primarily relies on different global poolings using various inputs from non-weighted features to global context vectors for channel transformation. This approach ensures generated weights will be more resilient to noisy labels on medical images.

Local interaction may benefit from previous approaches with a bottleneck structure. Firstly, in the original channel transformation of the GC block, the main components of the bottleneck are 2D convolutions. Hence, during the operation of this structure, it generates features with size W×H×C/r for shrinking the number of channels, where *r* is the ratio. This operation results in dimensionality reduction and causes the problem of information loss. Secondly, due to the main operation of 2D convolution, the time complexity of this convolution is $O(N^{2}K^{2})$ [48], indicating the high computation cost and slow inference, where *N* is the size of features and *K* is the kernel size. In contrast, the local interaction creates features with size 1×1×C so that the number of channels *C* does not change during the capturing, aggregating, and generating attention weights. Hence, it can address the problem of dimension reduction better than the previous bottleneck structure. Moreover, the operation of local interactions mainly uses 1D convolution to generate weights; these convolutions have a time complexity of O(NK) [48]. Also, the operation on 1D convolution costs fewer required parameters than 2D convolution. As a result, the proposed process effectively reduces complexity without shrinking dimensionality while also producing more precise attention weights. Additionally, choosing features from Equation (5) can adaptively determine the most important ones for channel attention weight generation using a very large kernel size. This approach maintains robust visual feature maps and generates improved attention maps, which enhance the DL models’ ability to withstand noisy labeling by combining data from two distinct global pools. As a consequence, the whole process of the proposed local interaction for global context vectors can enhance their ability in a more effective way than the bottleneck structure, assisting the DL models better under medical image conditions. Figure 4 indicates our design structure of the proposed equations above of local interaction with global context modeling. This combination is then called Global Context and Local Interaction (GCLI) module A. In summary, the proposed method handles noisy labels on medical images by first introducing two branches of global pooling, each focusing on specific information to generate characteristic sequences. Next, these results are then aggregated with size 2×1×C and passed through a 1D convolution with a large kernel size to adaptively choose appropriate weights. These parts are the most important steps that assist the module of incorporating different unique information to create robust weights that can deal with noisy labels effectively before being used to form the local interactions between channels for final weight generation.

In Figure 4, to gain a better understanding of GCLI module A implementation, the global context modeling section is shown in Equation (2). The Global Avg Pool and Global Max Pool are the global pooling proposed in Equations (3) and (4): the Global Avg Pool will consider the conventional channels for addressing noisy labels, while the Global Max Pool will capture the most noticeable features among global context weighted channels, which are outcomes of modeling global context vectors. The outputs of these two pooling are the two separated sequence channels with sizes 1×1×C, respectively. Then, they are aggregated and passed to a 1D convolution with a large kernel size of 7×7 to choose suitable channels from two different global feature descriptors. The chosen channels are then activated in a higher complex space using the SILU activation function, as shown in Equation (5). This step will produce a single set of selected channels, which will be sent to the last 1D convolution with a 3×3 kernel size to create channel weights, as shown in Equation (6). Finally, the sigmoid function then activates the attention weights; the result of this process is then multiplied with the original input channels to create the final weighted channels.

### 3.2. Partial Attention Strategy for Suppressing Weighted Redundancies in the GCLI Module

The process of mapping features to more complex spaces during the feature extraction phase aims to introduce more abstract features, which should enhance the overall detection ability of DL models under sophisticated labels. However, the deeper the extraction layers, the more irrelevant features tend to appear. So, in order to analyze the weighted redundancies in the proposed GCLI module A, the partial attention strategy is introduced and used, which is based on the idea of partial convolution from earlier work [43]. This previous study explored the redundancies in convolution and proposed the partial convolution, which uses only a small portion of channels as the input of convolution instead of considering every single one. However, when the original implementation is used on the GCLI module A, which only uses one quarter of the input channels for weight generation, the desired output is not reached. Hence, we increase the portion to one half, as the previous suggested ratio in the scenario of the attention mechanism is insufficient, as too few features are used for the attention weights procedure, making the result not generalized. As a consequence, the GCLI module with partial attention strategy was applied, calling the GCLI module B.

**Figure 5:**
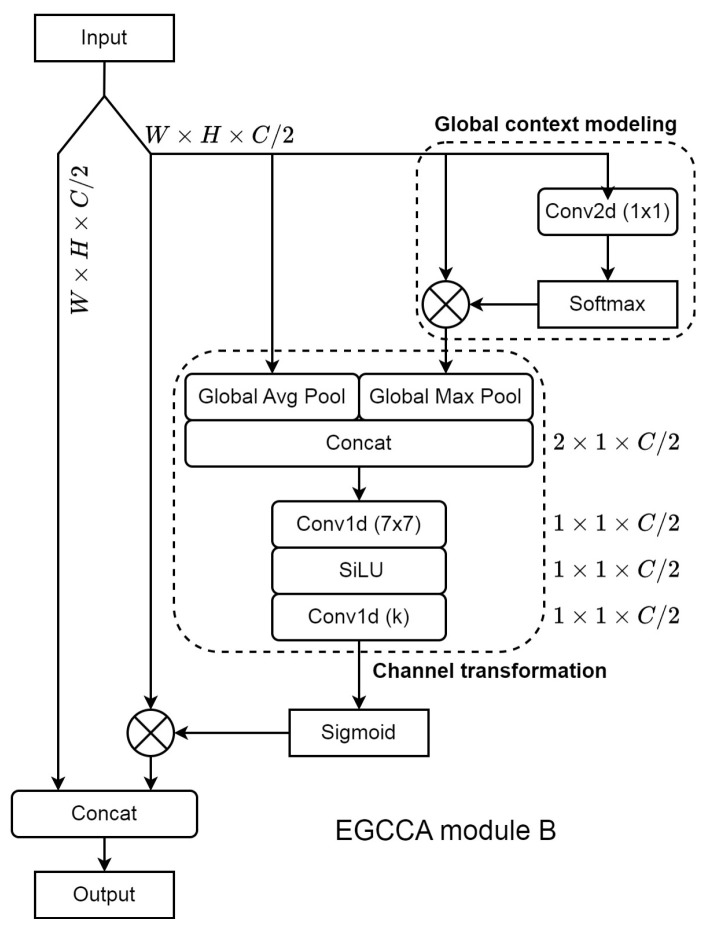
The structure of GCLI module B. This structure is the same as the GCLI module A except for having the partial attention strategy applied with one half of the channels used for weight generation. The approach aims to mitigate the effect of weighted redundancies caused by the attention mechanism.

The structure of the redundancies suppression GCLI module B is described in Figure 5. In detail, to understand our method, the original input with size W×H×C is then split into two separate sequences: the non-interacted sequence and the interacted sequence with similar size W×H×C/2. The module would leave the non-interacted sequence untouched, while it would feed the interacted sequence forward to the GCLI module to generate attention weights. When Equations (2)–(6) are used with the partial attention strategy, the sequence channels’ sizes are cut in half: from 1×1×C and 2×1×C to 1×1×C/2 and 2×1×C/2. Applying this strategy not only enables the GCLI module to conduct experiments and analyze the partial attention effect but also leads to a slight reduction in extra parameters and memory cost. The partial attention strategy leads to a redesign of the GCLI module’s final output, which concatenates with the non-interacted sequence after applying the generated attention weights to the features.

### 3.3. The Proposed Positions of GCLI Modules in Deep Learning Architectures

Previous works introduced applying the proposed attention modules into various classical CNNs [15,29,30,31,32,33]. However, different attention modules were placed at different positions in the architectures. Hence, some may perform better than others at specific positions. Therefore, we propose GCLI modules that occupy multiple positions in various classical CNN architectures, enabling us to evaluate the module’s performance across different phases of the architecture. The main focus of these proposed networks is anomaly classification and object detection on medical images.

First, several noticeable CNN architectures have been selected, including MobileNetV2, MobileNext, and ResNet, for the integration of GCLI modules in classification tasks. While MobileNetV2 was mainly proposed for DL to perform applications on low platforms, the improved version, MobileNext, was introduced as the better balance between accuracy and inference speed. ResNet is considered the most noble CNN architecture, featuring residual connections. These architectures are introduced briefly as follows:

- MobileNetV2 [49]: the unique CNN that is designed most to fit with the mobile application. The architecture is the combination of residual connections between bottlenecks, which are called inverted bottleneck structures. The GCLI module is being added at the end of every inverted bottleneck structure in order to capture most of the information extracted by specific blocks and create the attention weights precisely.
- MobileNext [44]: the next version of the MobileNet family, in which it exploits the information loss from the previous inverted bottleneck structure. Hence, the authors proposed to flip the entire previous bottleneck structure to the new novel design called sandglass block, which is able to transform features to the higher dimensions. To further strengthen the architecture, the GCLI module is fused right after at the end of each sandglass block.
- ResNet [50]: the CNN that first introduced the residual connection in order to prevent the problem of vanishing gradients in previous architectures during optimization in the training phase. Similar to MobileNext, the GCLI module is added right after every residual block in the architectures.

**Figure 6:**
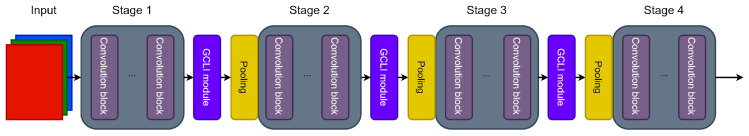
Proposed positions of GCLI module in CNN architecture. The proposed GCLI modules are suggested to be fused at the end of every block in the extraction phase.

For better observation, the proposed positions of GCLI modules in many CNN architectures can be illustrated in Figure 6. In this figure, the original design of several CNNs consists of several stages; each of them have a unique block that is used for feature extraction. In order to evaluate the effect of the proposed attention module in focusing on extracted information, the GCLI modules are suggested to be placed at the end of each block before the final global pooling in the architectures to enhance the quality of the feature maps extracted from these blocks.

**Figure 7:**
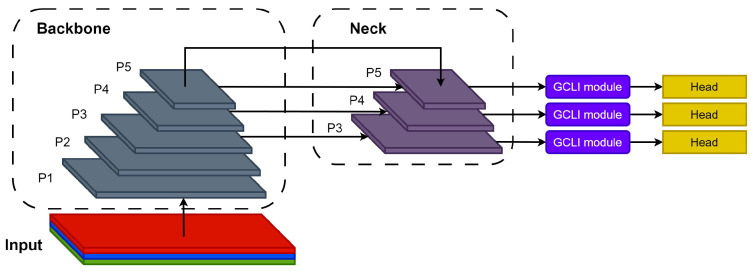
Proposed positions of GCLI module in YOLO architecture. The GCLI modules are suggested to be fused at the end of every scale in the aggregation phase of the multi-scale detection framework.

Second, in order to evaluate the GCLI modules in object detection tasks on medical images, the ResNet version of 50 layers with GCLI modules attached is then used as the backbone of RT-DETR [51], which aims to evaluate the performance of GCLI modules during the extraction phases. Our work further fuses solely the GCLI module at the end of every scale in the multi-scale detection framework of YOLOv8 [52] in order to evaluate its performance on the final stage of aggregation phases of the one-stage detector, YOLOv8. Figure 7 indicates the proposed positions of GCLI modules in YOLO architecture.

By carrying out different configurations of GCLI modules for specific types of architectures, the aim of the study is to evaluate the performance and behavior of GCLI modules in several scenarios: from specific computer vision tasks to different architectures. These proposed networks are then evaluated on three noisy datasets, including the Chaoyang dataset [53], the GRAZPEDWRI-DX dataset [6] and the VinDR-CXR dataset [54].

## 4. Experiment

### 4.1. Experimental Datasets

The three medical imaging datasets indicate the large amount of noise, including overlapped objects, various sizes, and classes that share similar features. The challenges put some considerable burdens on the model’s performance. Below is our brief description and the ratio of training set, validation set and test set of each dataset:

Chaoyang dataset [53]: the tiny yet noisy dataset that is constructed in the real scenario and comprises colon slides with the size fitted to 512×512. There are 6160 images with four classes, including normal, serrated, adenocarcinoma, and adenoma. These classes indicate similar features like shape, color, and patterns that may be misleading to normal approaches. The original dataset was already split by authors and consists of 4021 images for training and 2139 for testing. In this study, another 20% of the training set has been split for the validation set. As a result, there are 3217 images in the training set, 804 images in the validation set and 2139 images in the test set. The initial approach from the authors of this dataset was a postprocessing method that used denoising heads that automatically adapt to different levels of noisy labels. While this method is effective for addressing small and noisy datasets, it adds some considerable complexity to the whole architecture.

**Figure 8:**
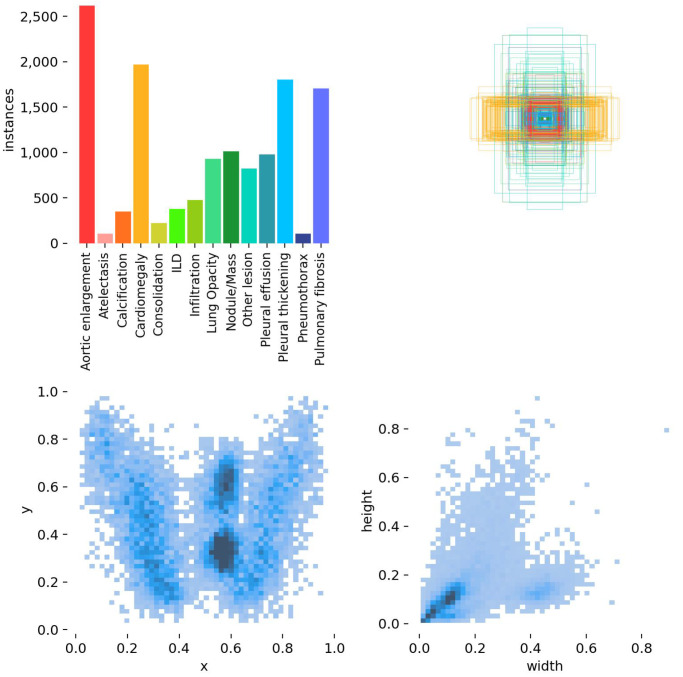
Distribution of location and size of objects in the VinDR-CXR dataset. Highly dispersed sizes and shapes of objects can be observed.

VinDR-CXR dataset [54]: the medium-scale dataset for detecting and locating anomalies on chest X-ray images. The dataset consists of 18,000 images for training and 300 images for testing. Moreover, Figure 8 shows that it consists of a lot of overlapped labels and similar classes or small objects that would increase the difficulty of model detection performance. However, due to the unavailable labels for the test set, this set is not used in evaluation. Furthermore, as this dataset is used for object detection tasks, any images with a “normal” label are discarded. Eventually, there are 4394 samples. These images are then scaled to 1024×1024 and split into three sets as follows: 2636 images for training, 659 images for validation, and 1099 images for testing. Previous work [55] that evaluated this dataset for object detection has illustrated low performance due to the intense effect of noisy labels.

**Figure 9:**
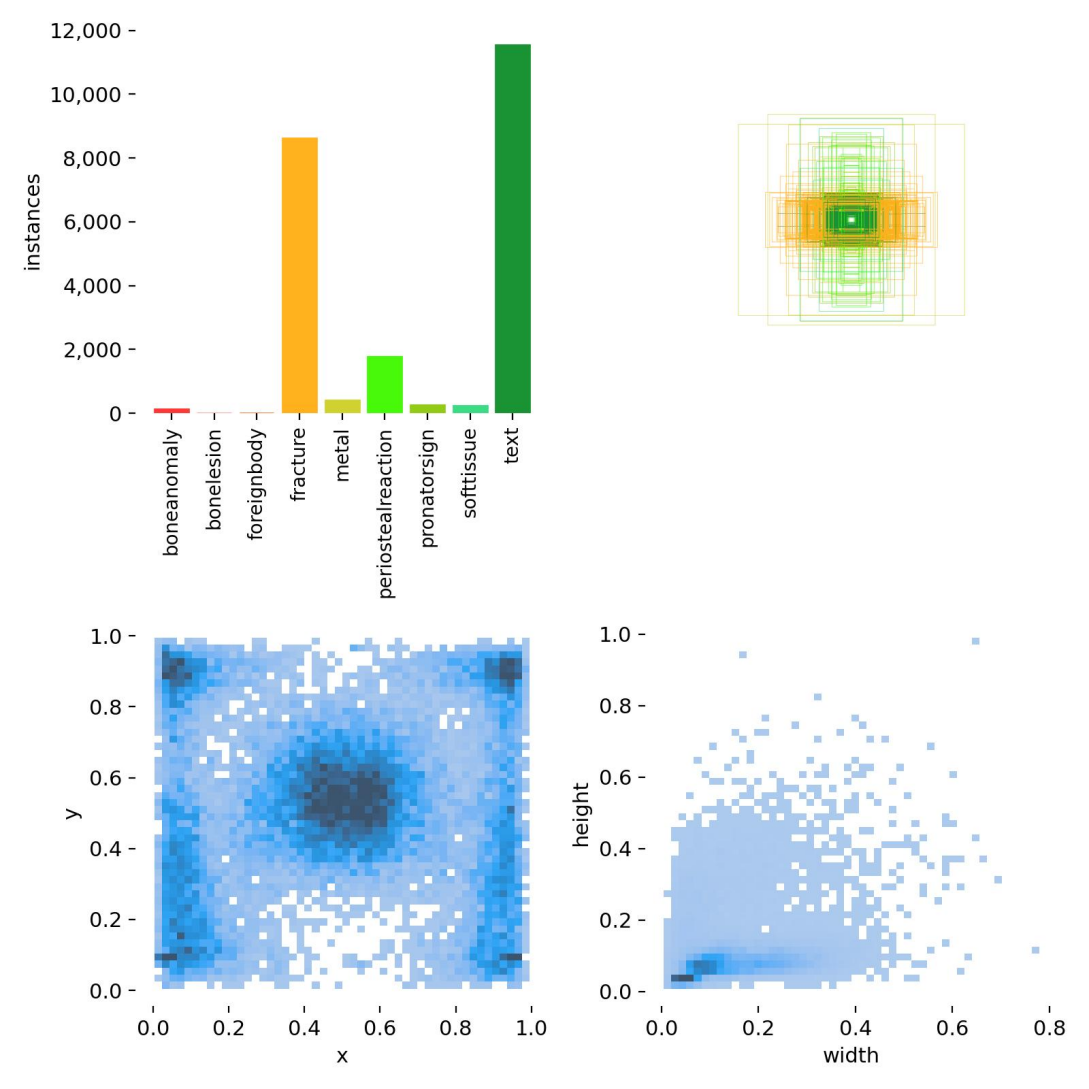
Distribution of location and size of objects in the GRAZPEDWRI-DX dataset. Highly imbalanced classes with various size objects can be observed.

GRAZPEDWRI-DX dataset [6]: the large-scale dataset for wrist trauma detection on X-ray medical images. There are 20,327 images with various sizes and up to 11 classes, including tags and other descriptions. This dataset consists of different brightness or noise on the image and even similar targeting objects as indicated in Figure 9. However, due to the unavailable training set, validation set, and test set, our study proposes the splitting as follows: 9873 images for training, 5311 images for validation, and 5143 images for testing. More images used for testing may increase the generalization of the final result of different models.

In order to understand better the splitting ratio of the three introduced datasets, Table 1 below indicates the number of images in the training set, validation set, and test set of each dataset.

### 4.2. Experimental Metrics

In order to evaluate the proposed GCLI modules with several computer vision tasks under the effect of noisy labels on medical images, the following metrics are used:

Parameters: the number of trainable weights in the DL models required to perform the training and predicting tasks. This metric is mainly used to evaluate the complexity cost of the model.

Accuracy: the metric that measures the proportion of correct predictions generated by DL models out of all predictions. This kind of metric is commonly used in classification tasks; the formula of accuracy can be described in Equation (7).

Precision: the metric that illustrates the ratio of correctly predicted positive observations to the total predicted positive observations. This metric is applied for evaluation in object detection by using the ratio of correctly detected objects to all detected objects. The final output of the metric is the ratio of correct labels predicted by the DL models. The high precision illustrates that DL models rarely detect objects where none exist. The formula of precision can be described in Equation (8).

Recall: the ratio of correctly predicted positive observations to the actual number of positive observations. The metric is applied in object detection by using the ratio of correctly detected objects to all objects that exist in the ground truth. The result of the equation is the ratio of correct labels predicted by the DL models. The high recall indicates that DL models successfully detect most of the actual objects in the image. The equation of recall is described in Equation (9).

F1 score: the harmonic mean of precision and recall; this metric is primarily used to evaluate datasets with imbalanced classes. In case both precision and recall are important, the F1 score is considerably suitable. The formula of the F1 score is illustrated in Equation (10).

Area Under the Curve (AUC) score: the metric that evaluates the area under the Receiver Operating Characteristic (ROC) curve, which plots the True Positive Rate (TPR) and the False Positive Rate (FPR) at various thresholds. The range of this measurement is from 0 to 1, where 1 is considered the best model, while 0 is worse than random.

(7) $$Accuracy=\frac{TP+TN}{TP+TN+FP+FN}\times 100\%$$

(8) $$Precision=\frac{TP}{TP+FP}\times 100\%$$

(9) $$Recall=\frac{TP}{TP+FN}\times 100\%$$

(10) $$F1=2\times \frac{Precision\times Recall}{Precision+Recall}$$

In Equations (7)–(9), TP is true positive, TN is true negative, FP is false positive and FN is false negative.

Mean average precision (mAP): a metric used widely in object detection tasks to evaluate the DL model’s precision across different Intersection over Union (IoU) thresholds and classes by considering both precision and recall, which is illustrated as:

(11) $$mAP=\frac{1}{N}\Sigma_{j=1}^{N}AP_{j}$$

where

(12) $$AP=\Sigma_{n}\left(R_{n}-R_{n-1}\right)\times P_{n}$$

In Equation (11), *N* is the total number of classes and $AP_{j}$ is the average precision for class *j* at a specific IoU threshold. In Equation (12), Rn, $R_{1}$, $R_{n-1}$ are the recall values at different thresholds and $P_{n}$ is the precision at each recall threshold $R_{n}$.

In this study, the precision, recall, F1 score, and AUC score are computed, and we used the macro-average to average each class. In addition, the mAP@0.5 and mAP@[0.5:0.95] are used for the object detection task. For convenience, the rest of this paper will refer to mAP@0.5 as mAP50 and mAP@[0.5:0.95] as mAP50–95.

### 4.3. Experimental Setup

In order to evaluate the performance and behavior of GCLI, the first ablation study is conducted, which indicates thoroughly the process of how GCLI is made from the original GC block with various experiments and the final version of the GCLI modules. The second experiment is carried out using GCLI modules A and B to evaluate their performance with other noticeable attention modules such as CBAM, ECA, simAM, CA, and GC. These SOTA attention modules are added at the proposed positions of GCLI modules in the DL architectures. From this section to the rest of the article, GCLI module A is referred to as GCLI-A, and GCLI module B is referred to as GCLI-B for convenience in table illustration. All of the experiments carried out in this study are conducted with Pytorch 2.4.0 and CUDA version 12.1 on a single machine with the following system configuration: CPU Intel Xeon Silver 4208 with 98 GB RAM and GPU RTX A100 40GB VRAM to accelerate. All DL models are trained from scratch without using any pretrained weights for a fair comparison.

For the classification task, all of the DL models used the same hyperparameter value as indicated in Table 2, as the epoch is set as the same as the hyperparameter that was used to conduct the experiments in the original paper of the Chaoyang dataset, while both training and testing use the same input size of 512×512.

For the object detection task, all of the DL models used the same hyperparameter value as indicated in Table 2. However, the input size for both training and testing on two datasets, VinDR-CXR and GRAZPEDWRI-DX, is different in order to evaluate the performance of GCLI and other attention modules with various sizes of images. The setup of the input size is indicated in Table 3.

### 4.4. Ablation Experiment Analysis

The ablation study is carried out on both classification and object detection to thoroughly understand the impact of each component. This section was prepared and analyzed carefully to examine the process of how the change is made and its effect from the original to the final version of the GCLI modules. Our study considers the two most noticeable models for integrating and testing the impact of each component in GCLI modules, including ResNet34 on the Chaoyang dataset for classification and YOLOv8 on the GRAZPEDWRI-DX dataset for object detection. Each model is sequentially evaluated in terms of performance and the number of parameters. Table 4 illustrates the ablation study of each component in the classification task on the Chaoyang dataset, while Table 5 shows the ablation experiment in the object detection task on the GRAZPEDWRI-DX dataset.

**Figure 10:**
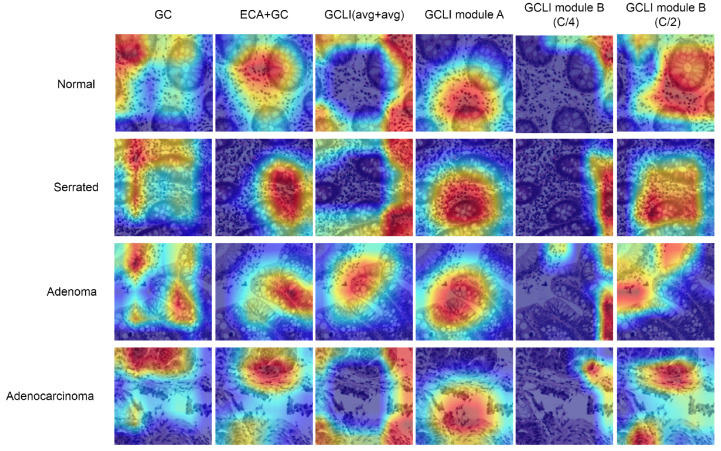
Attention maps of different modifications on the Chaoyang dataset. The final version of GCLI modules, including GCLI-A and GCLI-B (C/2), can lead the models to consistently focus on the wider important areas rather than shattered or weaker regions compared to others.

**Figure 11:**
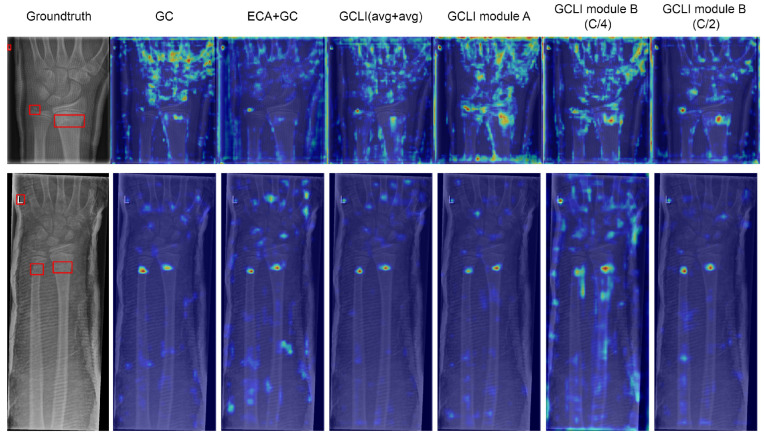
Attention maps of different modifications on the GRAZPEDWRI-DX dataset. GCLI module A and GCLI module B (C/2) can reduce the risk of weighted redundancies, leading to a more accurate location of targeting areas and suppressing other non-relevant regions.

In Table 4 and Table 5, the GC is the original global context block, and the ECA+GC is our first attempt to incorporate local interaction with global context modeling. The GCLI (avg+avg) is the approach that utilizes the two global average poolings for the non-weighted channels and weighted channels. While GCLI module B (C/4) represents our initial attempt to implement the original partial attention strategy, which utilizes one fourth of the number of channels for weight generation, our final solutions, GCLI modules A and B (C/2), successfully integrate local interaction with global context modeling and suppress weighted redundant information using one half the number of channels for weight generation. Initially, with the GC block in ResNet34, if being compared with the ECA module in further analysis section below, it can be seen that the GC block did not surpass the performance as desired. This observation suggests that the GC block is not the optimal solution when dealing with noisy labels. Therefore, our initial attempt involved combining the ECA module with the GC block, essentially replacing the bottleneck structure in the channel transformation of the GC block with the ECA module, creating the ECA+GC Block. As a result, the accuracy is increased from 77.79% to 80.5%; this shows that the bottleneck structure greatly limits the performance of global context modeling. By applying naive local interaction to learn important channels that prevent any dimensionality reduction, the global context modeling module is able to reach higher accuracy. Furthermore, the local interaction, primarily using 1D convolution, reduces the parameter from 21.33 to 21.28 million, indicating a slight improvement in the model complexity. However, with the same ECA+GC block in YOLOv8, the performance is slightly decreased from 48.6% to 48.2% mAP50; this has failed our expectations and led to further analysis. In Figure 10, under noisy labels like the Chaoyang dataset, the GC block tends to focus on irrelevant information, which may be misleading to model prediction, while the ECA+GC block tends to direct the model to the more important features on the images. Nonetheless, in Figure 11, while the GC block could introduce important features to the model, it also adds lots of redundant features to the images. The ECA+GC block not only significantly reduces redundant features but also weakens important information. This can be understandable as ECA mainly used global average pooling as its global information descriptor; this unique pooling is widely known for its ability to suppress noises. As a result, its strong influence unintentionally reduces useful information.

Our next approach aims to reduce its undesirable behavior by introducing a second branch from non-weighted channels and passing it through another global average pooling. The output of two branches is then aggregated and passed to a 1D convolution to select important channels before being used for weight generation. This modification was called GCLI (avg+avg), leading to the moderate increase in accuracy from 80.5% to 81.95% and mAP50 from 48.2% to 49.0%. At this phase, in Figure 10, the module starts to introduce bordering information, while in Figure 11, the module retained more useful information after filtering irrelevant information. At this point, it is clear that the section on selecting characteristic channels has proven its capability. Moreover, because global context modeling is a critical component in adding more complex interactions between spatial features, further filtering irrelevant feature pooling only made the useful information weaker. As a result, global average pooling is replaced with global max pooling to retain more highlighted information from global context modeling, while non-weighted channels are used for global average pooling to suppress the noisy label effect. With these modifications, our first GCLI module A is introduced, the performance of the ResNet34 is able to increase from 81.95% to 82.46%, and the mAP50 metric of YOLOv8 is raised from 49.0% to 49.5%. However, when looking at Figure 10, it is indeed that the module then directs the model to the more useful information, but in Figure 11, while it introduces more robust features at targeting location on the images, this module also struggles with high redundancies even with the assistance of global average pooling. Hence, these observations have answered our question: the global context modeling with local interaction has the potential risk of generating weighted redundancies. To address the problem, adopting the idea of work [43] with partial convolution for addressing the problem of redundancies in convolution, our analysis further exploits this idea and develops a partial attention strategy. In the previous work, the author suggested using one fourth of the channels for convolution. Our first experiment applies this idea to the GCLI module. As a result, the accuracy of ResNet34 is decreased slightly from 82.46% to 82.28%, and the mAP50 of YOLOv8 is down moderately from 49.5% to 48.7%. Hence, these figures indicate that the original use of one fourth (C/4) of the channels for generating weights does not achieve our desired output. Further attention maps from Figure 10 and Figure 11 illustrate that the model concentration is insignificant, misleading spatial information. Our observations indicate that the previous portion is insufficient for the operation of the attention mechanism. Therefore, the portion is increased to one half (C/2) in our proposed approach; this modification is our final version, the GCLI module B. As a result, ResNet34 is able to increase its accuracy from 82.28% to 82.51%, and the YOLOv8 performance is raised from 48.7% to 52.1% mAP50, which indicates the highest performance among each category. Furthermore, in Figure 10, the model starts to consider an extremely wide range of important features compared to previous modifications, while in Figure 11, the module is able to introduce more robust relevant features with a greater ability to suppress weighted redundancies. Additionally, the implementation of the local interaction and partial attention strategy not only enhances the performance of global context modeling but also results in a reduction in the number of parameters and memory consumption. Ultimately, this ablation study demonstrated that our approach accurately integrated local interaction with global context modeling, and the partial attention strategy has proved its effectiveness in various scenarios involving noisy or small datasets.

**Figure 12:**
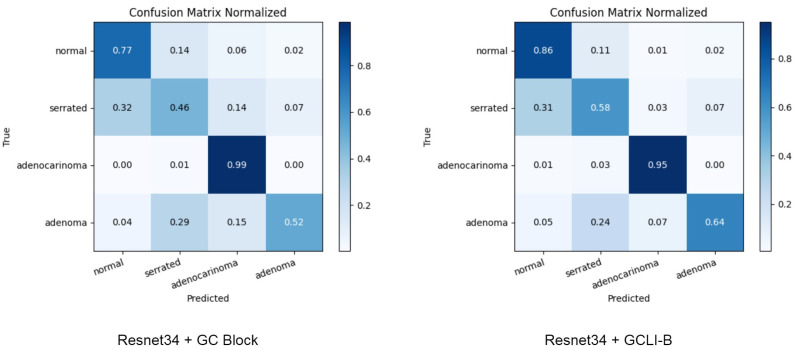
Confusion matrix of ResNet34 with GC block and GCLI module B. GCLI module B can assist the model with achieving higher performance with other noisy labels compared to the GC block.

To further understand how the final version of our proposed attention module, the GCLI module B, improved over the GC block, the confusion matrix is conducted and illustrated on the classes of the Chaoyang dataset in Figure 12. Both attention modules show their remarkable high accuracy in adenocarinoma class, which are 99% by GC block and 95% by GCLI module B. Despite the marginal decrease in this class, our proposed GCLI module B illustrates its significant performance in addressing other noisy classes. For instance, the proposed module has the higher accuracy in the serrated, which is 58% compared to 46% of the GC block. In terms of adenoma class, GCLI module B is able to achieve a moderate improvement over the GC block, which is about 12% higher in accuracy. In addition, in the normal class, while the GC block only achieves 77% in accuracy, our proposed GCLI module B is capable of reaching up to 86% accuracy. In conclusion, these figures indicate that noisy and small datasets significantly decrease the attention weight’s ability created by the original GC block, while our proposed GCLI module B can address this problem effectively. Hence, GCLI module B can assist the DL models by improving their classification ability in noisy environments, particularly classes that share similar features and patterns.

### 4.5. Comparison Experiment Analysis

In this section, the experiments test both complexity, including parameters and inference time, and performance on classification and object detection tasks with other SOTA attention modules using the introduced noisy label datasets. Firstly, Table 6 illustrates the number of required parameters for each of the attention modules.

As Table 6 indicates, ECA and simAM are the two modules that consume the fewest extra parameters. While ECA only focuses on channel attention type, simAM is the 3D attention that considers both channel and spatial features for generating attention weights simultaneously. To understand the concept behind these kinds of low parameter consumption attention modules, it is noticeable that ECA mainly relies on 1D convolution to construct attention maps, and simAM uses an energy function, which is similar to the spatial suppression mechanism [56], to create proper weights. Among other attention modules, the GC block has the most extra parameter consumption, adding up to 33,889 extra parameters, followed by CBAM with 32,866, and CA in third place with 25,648. Apparently, these attention modules have the ability to perform better attention weight generation. For instance, the CBAM generates attention weights by sequentially considering channel and spatial information; the CA shares the concept of 3D attention with simAM but requires more parameters; and the GC block primarily models the global context, which closely resembles the long-range dependencies in the self-attention mechanism. However, the high resource requirements of these attention modules pose challenges for integration into smaller DL models. Additionally, if increasing the number of channels is necessary for higher accuracy, these attention modules may add significant extra parameters, slowing down the inference speed of DL models. In spite of this, our suggested GCLI modules only need 530 extra parameters to allow channel transformation with global context vectors. This makes them nearly 64 times lighter than the dedicated GC block due to the proposed local interaction approach. Furthermore, GCLI module B requires only 146 extra parameters, making it approximately 3.6 times lighter than the GCLI module A and 225 times more efficient in terms of parameter consumption than the GC block. With this extremely low consumption of resources, our proposed GCLI modules are able to perform global context modeling ability or be used with a larger channel with a significant small trade-off in extra parameters.

**Figure 13:**
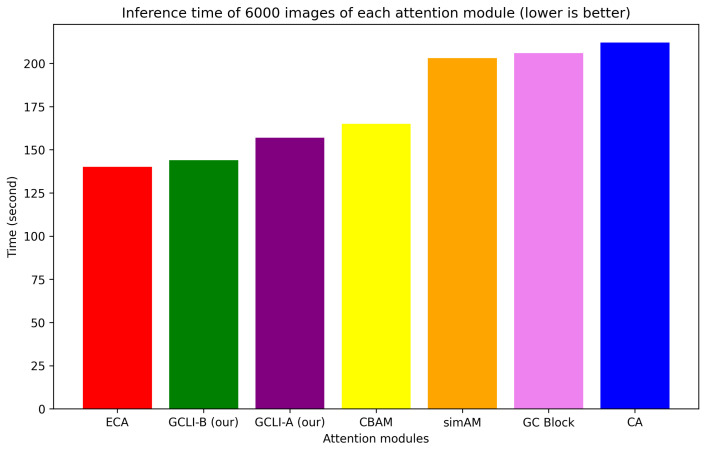
Total inference time of each attention module. The proposed GCLI module B has a very competitive inference speed compared to ECA, while GCLI module A is even faster than CBAM.

Secondly, Figure 13 illustrates the total inference time of each attention module in seconds.

From the illustration in Figure 13, it can be seen that while simAM is a zero-parameter consumption, it is one of the top three most costly in time execution that reach more than 200 s to process 6000 images. These figures show that even with minimal parameter consumption, the attention module can significantly increase computation costs. The other two are CA and GC blocks, which also require an even higher amount of time than simAM to execute on the same number of images. Despite the effectiveness of 3D attention models or global context modeling modules, which consider both spatial and channel features for attention weight generation, they incur significant costs in terms of extra parameters and execution time. ECA is the fastest attention module in the category, requiring only about 140 s for inference. However, this attention module only creates channel attention. Another robust attention module that can create attention weights for both channel and spatial features sequentially is CBAM, taking a moderately higher inference time than ECA, which is about 165 s. Our proposed GCLI modules are unique as they are capable of processing significant data quickly while gaining the ability to model global context. In detail, GCLI module A takes about 155 s to process 6000 images, and GCLI module B takes nearly the same amount of time as the ECA module. When comparing this result to the GC block, which is also capable of modeling global context, our proposed GCLI modules are able to execute at a rate faster than 30% while maintaining the same level of capability. Overall, the GCLI modules, incorporating local interactions, offer an efficient global context modeling solution that minimizes extra parameter consumption and computation cost. This makes them an ideal choice for many DL models that aim to integrate long-range dependencies with minimal complexity trade-off.

Next is the performance comparison on the classification task. First, the attention modules are integrated into MobileNetv2, MobileNext, and ResNet34 to train and test on the test set of the Chaoyang dataset. Table 7 illustrates the result of each attention module on DL models, where “-” is the baseline version of the DL models that is not integrated with any attention modules. As the table indicates, under the scenario of noisy label effect, many architectures with attention modules tend to perform worse than the baseline model. For instance, simAM with MobileNetV2 only obtains 71.48% accuracy compared to 74.05% of the baseline version; this figure is the lowest one in the category. This indicates that this attention mechanism is being affected by the noisy labels, making the module introduce irrelevant features that are misleading to the model prediction. Other attention modules such as CBAM and CA both can increase the MobileNetV2 performance from 74.05% accuracy to 78.58% and 80.69% and boost the accuracy of ResNet34 from 79.61% to 79.85% and 80.22%, respectively. It is proved that the combination of channel attention and spatial attention in CBAM or coordinate attention in CA is effective against the noisy labels. However, these attention modules are not lightweight, as they put in a considerable amount of extra parameters. For instance, in MobileNext, CBAM adds up to 3.37 and CA adds up to 2.49 million parameters compared to 1.92 of the baseline version. With GC block, the ability of global context modeling is not performed well under many CNNs, such as MobileNext and ResNet34, where the attention module reaches the worst performance compared to the baseline. Eventually, our proposed attention module GCLI module A can easily boost the performance of each architecture effectively without nearly adding any extra parameters. Moreover, GCLI module B can assist MobileNetV2 in reaching up to 80.27% with nearly the same number of parameters compared to the baseline model. Furthermore, in MobileNext, GCLI module B can help the model achieve 79.52% accuracy, which is the highest figure among this category with a slightly increased number of parameters to 1.93 million. The highest performance of all categories belongs to ResNet34 with GCLI module B attached, which reaches up to 82.51% accuracy while maintaining the same resource consumption. With these observations, it is clear that the global context modeling ability in the GC block is being restricted remarkably by the bottleneck structure. Our proposed GCLI module, which primarily utilizes local interaction, significantly enhances the performance of the global context modeling module. The proposed approach is not only able to reduce complexity but also effectively address the problem of noisy labels in medical datasets. Furthermore, the introduced partial attention strategy demonstrates its ability to aid the GCLI module in reducing the impact of weighted irrelevant information resulting from global context modeling. As a result, the GCLI modules easily outperform other SOTA attention modules in helping models determine where to focus when faced with noisy labels on medical images. To conclude, the local interaction is eventually proved to best fit with global context modeling rather than the bottleneck structure, and the partial attention strategy can play a vital role under noisy labels in medical imaging datasets.

To understand how our suggested modules can improve model performance compared to the newest studies, other latest studies are added to compare in Table 8, which evaluates the performance of both our GCLI modules attached to ResNet34 with other studies on the Chaoyang dataset. Apparently, our work is better than the original study of the Chaoyang dataset [6] in terms of recall and F1 score metrics, which are 76.56% and 75.80%, respectively, while the original study has higher accuracy. Moreover, the original work requires another postprocessing filter named noise suppressing and hard enhancing (NHSE), which were trained additionally for 40 epochs, leading to a sophisticated architecture. As opposed to that, our proposed GCLI modules can be fused as a part of an architecture that is beneficial to model flexibility, resulting in improving the model’s performance without adding any extra filters. In the study [57], the Vision Transformer (ViT) architectures were used and trained for 100 epochs to obtain 82.52% and 81.25% accuracy, respectively, with the help of a self-supervised auxiliary task (SSAT). The accuracy achieved by the ViT-T with SSAT was only slightly higher than that of our approaches. However, by using extra stages to achieve better performance, these methods may add more computational cost, which may not be an optimized trade-off. In conclusion, our proposed GCLI modules can enhance architectures to achieve highly competitive performance without the need for additional stages or postprocessing filters, thereby enhancing model flexibility and optimizing the trade-off between complexity and performance. Furthermore, unlike the original self-attention mechanism in the ViT architecture, our proposed GCLI modules can still have a similar ability to capture long-range dependencies and adaptively assist various DL models in achieving high accuracy at small complexity and computation cost.

Further experiments are also carried out for object detection tasks with the aforementioned datasets. Table 9 illustrates the performance of RT-DETR and YOLOv8 with attention modules on the medium-scale VinDR-CXR dataset.

**Figure 14:**
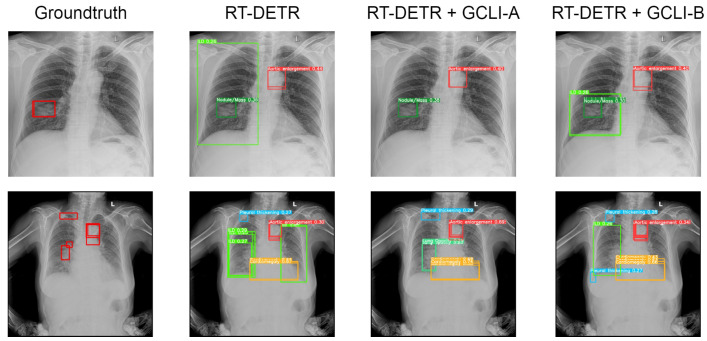
Several results of RT-DETR models on the VinDR-CXR dataset. The GCLI modules can decrease the misleading predictions of the model effectively and further lead to more potential targeting regions.

**Figure 15:**
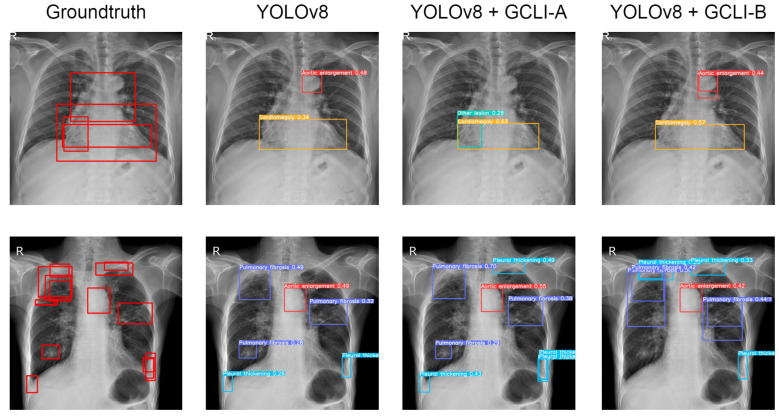
Several results of YOLOv8 models on the VinDR-CXR dataset. Both GCLI modules are able to address the noisy labels with various object sizes that the baseline model fails to adapt.

As Table 9 shows, with many overlapped objects on medical images, the architectures tend to perform worse. In terms of RT-DETR, only models with ECA, CBAM, and GCLI module A attached are able to surpass the baseline model. While ECA assists the model in reaching a higher mAP50, which is 10.3%, GCLI module A is able to increase the model’s recall, which is 27.9%. These two attention modules do not add much complexity to the models. However, GCLI module B is not the best option in the scenario of overlapped objects on medical images, as it only achieves 9.0% mAP50. In terms of YOLOv8, most of the attention modules tend to boost the performance of the architecture as they both reach higher performance compared to the baseline model. The GC block is the one that assists the model in achieving the highest mAP50 among attention modules in the category, which is 18.2%. However, this attention module introduces significant additional parameters to the architecture. Instead, our proposed GCLI modules A and B are able to boost the AP50 of YOLOv8 to 16.3% and 17.8%, respectively, resulting in GCLI module B standing in the second position of mAP50 without adding many extra parameters. From the results of the GCLI modules in both DL models, it can be concluded that under the effect of overlapping objects on medical images, GCLI modules may not be the best fit for handling this kind of noisy label. However, they can still increase model performance with a lower cost in complexity. Furthermore, the impact of attention modules varies depending on whether they are placed in the extraction or aggregation phases. In the extraction phase, GCLI module A typically outperforms GCLI module B and vice versa. Eventually, the partial attention strategy also indicates its benefits, especially if it is applied for the GCLI module that is placed at the aggregation phases rather than the extraction phases. Figure 14 and Figure 15 show the detection results for the baseline models RT-DETR and YOLOv8 as well as the changed version with our proposed GCLI modules attached. As illustrated, the proposed GCLI modules can address the problem of misleading objects better than the baseline models, introducing less redundant detection and providing higher precision performance.

Next is another object detection task with the large-scale GRAZPEDWRI-DX dataset. Table 10 illustrates the performance of RT-DETR and YOLOv8 with attention modules on the GRAZPEDWRI-DX dataset. The attention modules are fused in the ResNet50 as the backbone for the extraction phase of RT-DETR and at the end of every scale in the aggregation phase of YOLOv8. However, due to the extremely high computation cost of simAM in RT-DETR, the experiment cannot be conducted on time. Therefore, there are no reported data of simAM in this term.

**Figure 16:**
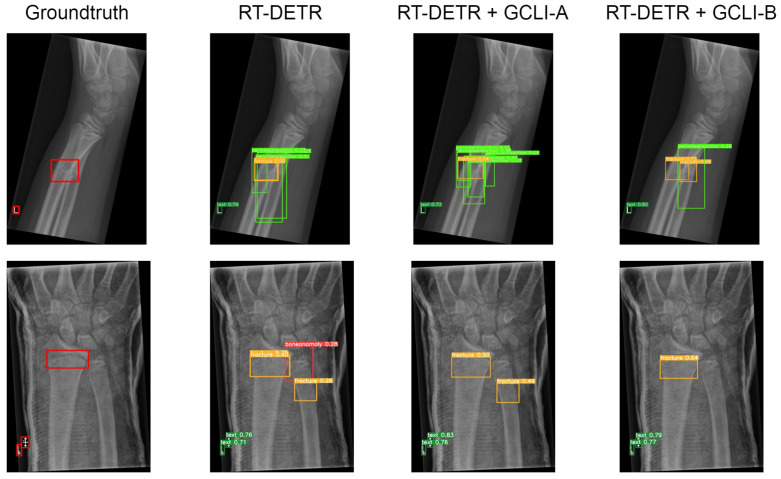
Several results of RT-DETR models on the GRAZPEDWRI-DX dataset. The proposed GCLI modules can assist the model effectively by introducing less misleading predictions under conditions of similar appearance between targeting regions and normal regions.

**Figure 17:**
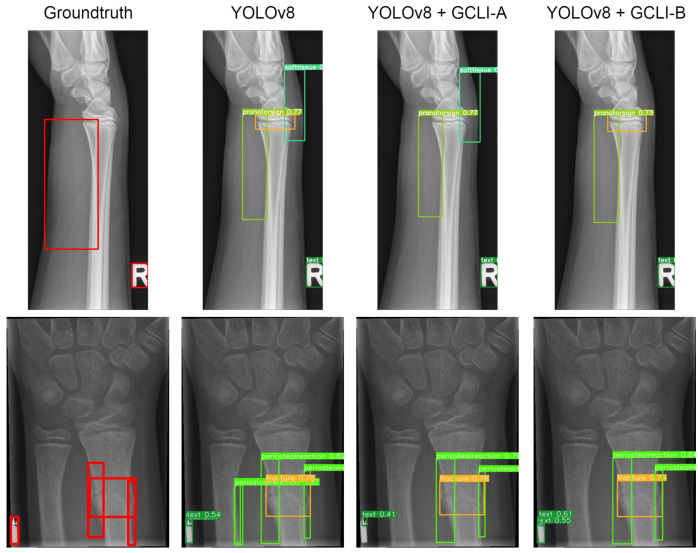
Several results of YOLOv8 models on the GRAZPEDWRI-DX dataset. The GCLI modules can boost the overall detection ability of YOLOv8 remarkably compared to the baseline version.

As Table 10 illustrates, some attention modules when fused with DL models tend to worsen the overall performance, such as ECA, CA, CBAM, and even GC block. In terms of RT-DETR, the DL model with the GC block attached maintains the same mAP50 as the baseline model, whereas the other ECA and CA only assist the model in reaching 42.1% and 41.8% mAP50, respectively. In terms of YOLOv8, both models with ECA and CBAM attached only achieve 48.6% and 48.9% mAP50, respectively. Even with simAM attached, the mAP50 of YOLOv8 is only sightly improved from 49.1% to 49.5%. These figures indicate that previous attention modules are struggling with noisy labels on medical images; while some tend to bring marginal improvement to the DL models, others may deteriorate the feature maps, resulting in a decrease in performance. On the other hand, our proposed GCLI modules effectively address this problem. For instance, GCLI module A enables the RT-DETR to reach the highest figure in mAP50, which is 43.9%, while GCLI module B assists the model in reaching the highest recall, which is 45.7% without adding many extra parameters. Moreover, in terms of YOLOv8, GCLI module B allows the model to reach the highest performance of all categories, which are 52.1% mAP50 and up to 32.5% mAP50-95. From Table 9 and Table 10, it is obvious that GCLI module A performs better than GCLI module B in RT-DETR architecture, and GCLI module B outperforms GCLI module A in the YOLOv8. The findings show that the GCLI modules add weighted redundancies more during the aggregation phases than the extraction phases. The partial attention strategy can effectively lessen the impact of weighted redundant features. Based on our observations of how two different GCLI modules behave, we can conclude that local interaction can greatly enhance the performance of global context modeling, outperforming bottleneck structures in assisting the DL model with noisy labels. Moreover, the experiments also indicate that the partial attention strategy can play a vital role in addressing noisy labels on many medical image datasets. In order to fully utilize the performance of GCLI module B, it is crucial to consider placing the attention module at the later phases of the architecture, while the extraction phases may be more suitable for GCLI module A. Figure 16 and Figure 17 show how well our GCLI modules address the noisy labels compared to baseline models. It can be seen that the proposed GCLI modules can guide the model to the more potential lesion areas, significantly providing less redundant predictions, especially in the case of GCLI module B. The results indicate that our GCLI modules can be a suitable option for improving model performance with a small trade-off in complexity to gain a better detection ability in the scenario of noisy labels of several medical imaging datasets.

## 5. Limitations

**Figure 18:**
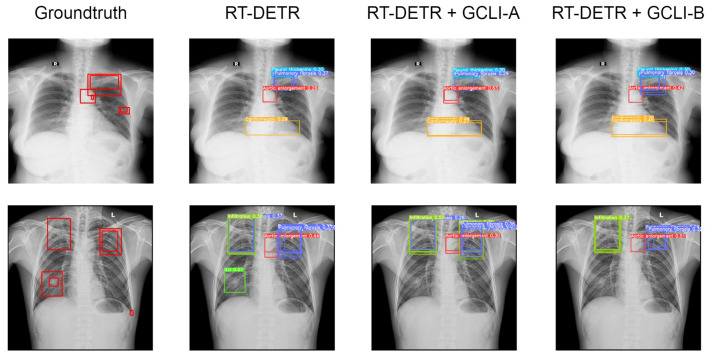
Several misleading detections caused by overlapped objects in VinDR-CXR dataset, indicating the limitation of the GCLI modules.

Despite the ability of the combined module between local interaction and global context modeling in addressing the problem of noisy labels in the medical dataset, the GCLI modules did not perform well under the scenario of overlapped objects in the VinDR-CXR dataset, while the baseline model tends to perform better in this term, as indicated in Table 9 and Figure 18, where several objects with highly overlapped areas may cause the model with GCLI modules to suffer compared to the baseline. Furthermore, the impact of the partial attention strategy varies depending on the characteristics of the dataset and the placement of the module within the architecture. Therefore, when overlapping objects appear densely, as in the VinDR-CXR dataset, or during the extraction phase, as in RT-DETR, this strategy does not perform optimally. As a consequence, future work to analyze the suitable positions of GCLI modules still needs to be carried out. In addition, the partial attention strategy is generally a passive method; it only utilizes a smaller number of channels for generating weights. This can accidentally reduce the effect of attention weights when dealing with complex classes, like the case in the VinDR-CXR dataset with overlapping objects. Hence, it is crucial to carry out further work with other solutions to deal with weighted redundancies in attention mechanisms.

## 6. Conclusions

In conclusion, our work has introduced GCLI modules, which could be a better version of GC blocks that assist DL models in focusing on important features better under the noisy label effect. These unique modules utilize local interaction and incorporate global context modeling, which not only enhances the module’s ability to generate attention weights under the influence of noisy labels on medical images but also reduces complexity without dimensionality reduction. However, through experiments, we found that the GCLI modules exhibit weighted redundancies, which significantly hinder the performance of the global context ability. To address this problem, a unique partial attention strategy is proposed and applied to GCLI modules. Further analyses have shown that this unique strategy has the potential to effectively address the redundant weighted features of modeling global context vectors in the GCLI modules. As a result, our proposed GCLI modules outperform most of the SOTA attention modules on both classification and object detection tasks in medical imaging under the noisy labels effect. In classifying tasks, the proposed attention module is able to boost ResNet34 up to 82.5% accuracy, which is 4.72% more than the GC block on the Chaoyang test set. In the GRAZPEDWRI-DX test set, the YOLOV8 with the GCLI module attached achieves 82.1% mAP50, which is 3.5% more than the GC block. Moreover, these proposed GCLI modules are able to use up to 225 times fewer extra parameters and up to 30% faster inference speed than the GC block and other robust attention modules. Given the ability to replicate long-range dependencies capturing of self-attention in global context modeling, the study indicates the potential for further works with this type of attention mechanism due to the previous problems of channel transformation in GC block that have been addressed. Moreover, the current methods primarily concentrate on classification and object detection in medical images, thereby limiting their applicability to other computer vision tasks. Therefore, there is still potential for further work using GCLI modules to tackle other challenging topics.

## Figures and Tables

**Table 1:** Ratio of training, validation, and test set of each dataset.

Dataset	Training Set	Validation Set	Test Set
Chaoyang	3217 (52.2%)	804 (13.0%)	2139 (34.7%)
VinDR-CXR	2636 (59.9%)	659 (14.9%)	1099 (25.0%)
GRAZPEDWRI-DX	9873 (48.5%)	5311 (26.1%)	5143 (25.3%)

**Table 2:** Hyperparameter setup of DL models for both classification and object detection task.

Hyperpamaters	Classification	Object Detection
Epoch	30	70
Batch Size	16	16
Opimizer	AdamW	SGD
Learning Rate	0.001	0.01
Momemtum	-	0.937
Weight Decay	-	0.0005

**Table 3:** Image input size for training and testing of DL models for object detection task.

Dataset	Model	Input Size
VinDr-CXR	YOLOv8	1024
GRAZPEDWRI-DX		640
VinDr-CXR	RT-DETR	768
GRAZPEDWRI-DX		640

**Table 4:** Performance of each components in GCLI module on Chaoyang dataset (ResNet34).

Attention Module	Accuracy (%)	Parameters ($10^{6}$)
GC	77.79	21.33
ECA+GC	80.50	21.28
GCLI (avg+acg)	81.95	21.28
GCLI-A	82.46	21.28
GCLI-B (C/4)	82.28	21.28
GCLI-B (C/2)	82.51	21.28

**Table 5:** Performance of each components in GCLI module on GRAZPEDWRI-DX dataset (YOLOv8).

Attention Module	mAP50 (%)	mAP50-95 (%)	Parameters ($10^{6}$)
GC	48.6	30.7	43.7
ECA+GC	48.2	29.9	43.6
GCLI (avg+acg)	49.0	31.1	43.6
GCLI-A	49.5	30.7	43.6
GCLI-B (C/4)	48.7	30.7	43.6
GCLI-B (C/2)	52.1	32.5	43.6

**Table 6:** Parameters of each attention module with input and output channels are 512.

Attention Module	Attention Type	Parameters
ECA (k = 3)	Channel attention	3
CBAM	Channel and spatial attention	32,866
simAM	3D attention	0
CA	3D attention	25,648
GC	Global context and channel attention	33,889
GCLI-A (our)	Global context and channel attention	530
GCLI-B (our)	Global context and channel attention	146

**Table 7:** Performance of DL models with attention modules on the test set of Chaoyang dataset.

Attention Module	Model	Precision (%)	Recall (%)	F1 Score (%)	AUC (%)	Accuracy (%)	Parameters ($10^{6}$)
-	MobileNetV2	66.73	68.12	67.01	88.39	74.05	3.50
ECA		70.47	71.03	70.70	88.45	77.69	3.50
CBAM		71.68	70.88	71.09	88.67	78.58	3.56
simAM		68.36	65.41	65.45	88.42	71.48	3.50
CA		75.19	73.68	74.27	89.79	80.69	3.54
GC		69.09	65.68	66.03	87.52	77.18	3.53
GCLI-A (our)		74.43	72.92	73.51	89.55	79.56	3.50
GCLI-B (our)		74.09	72.41	88.77	89.62	80.27	3.50
-	MobileNext	71.29	67.84	69.00	88.03	77.32	1.92
ECA		71.47	72.62	71.84	91.32	78.21	1.92
CBAM		71.89	68.47	69.29	87.38	78.02	3.37
simAM		68.28	66.57	67.22	87.87	74.75	1.92
CA		70.56	67.83	68.61	87.78	76.76	2.49
GC		67.43	62.34	63.68	84.54	73.30	2.67
GCLI-A (our)		69.99	67.25	67.52	88.77	77.04	1.93
GCLI-B (our)		74.48	72.17	73.12	89.43	79.52	1.93
-	ResNet34	73.75	72.46	72.97	88.47	79.61	21.28
ECA		73.07	74.24	73.28	92.12	79.47	21.28
CBAM		74.82	69.98	71.54	88.18	79.85	21.37
simAM		72.35	71.13	70.94	89.95	79.42	21.28
CA		73.75	71.39	72.18	89.94	80.22	21.32
GC		72.90	68.57	69.92	89.83	77.79	21.33
GCLI-A (our)		78.65	72.43	74.27	94.12	82.46	21.28
GCLI-B (our)		77.86	75.80	76.56	94.19	82.51	21.28

**Table 8:** Performance comparison of proposed methods with other latest works in Chaoyang dataset.

Methods	Precision (%)	Recall (%)	F1 Score (%)	AUC (%)	Accuracy (%)
ResNet34 + NHSE [6]	78.33	75.45	76.54	94.51	83.40
ViT-T + SSAT [57]	-	-	-	-	82.52
ViT-S + SSAT [57]	-	-	-	-	81.25
ResNet34 + GCLI-A (our)	78.65	72.43	74.27	94.12	82.46
ResNet34 + GCLI-B (our)	77.86	75.80	76.56	94.19	82.51

**Table 9:** Performance of object detectors with attention modules on the test set of VinDR-CXR dataset.

Attention Module	Model	Precision (%)	Recall (%)	mAP50 (%)	mAP50-95 (%)	Parameters ($10^{6}$)
-	RT-DETR	11.5	25.7	10.1	5.4	42.7
ECA		12.3	22.0	10.3	4.7	42.7
CBAM		20.1	20.0	7.0	3.7	45.2
simAM		8.9	25.5	9.0	4.6	42.7
CA		8.4	24.5	8.7	4.5	44.6
GC		9.7	3.0	9.7	5.1	45.3
GCLI-A (our)		10.9	27.9	10.2	5.1	42.7
GCLI-B (our)		10.3	23.8	9.0	4.7	42.7
-	YOLOv8	20.1	29.3	15.9	7.6	43.6
ECA		18.4	30.8	16.2	8.0	43.6
CBAM		20.9	30.1	17.6	8.7	43.7
simAM		19.4	28.3	16.0	7.7	43.6
CA		24.0	26.0	16.6	7.8	43.6
GC		24.4	29.5	18.2	8.7	43.7
GCLI-A (our)		19.9	30.3	16.3	7.8	43.6
GCLI-B (our)		20.5	28.6	17.8	8.1	43.6

**Table 10:** Performance of object detectors with attention module on the test set of GRAZPEDWRI-DX dataset.

Attention Module	Model	Precision (%)	Recall (%)	mAP50 (%)	mAP50-95 (%)	Parameters ($10^{6}$)
-	RT-DETR	65.2	44.3	42.7	26.3	42.7
ECA		61.6	45.1	42.1	26.5	42.7
CBAM		70.0	43.7	43.5	27.5	45.2
simAM		-	-	-	-	-
CA		63.2	43.6	41.8	26.5	44.6
GC		64.3	44.7	42.7	27.0	45.3
GCLI-A (our)		65.2	44.8	43.9	27.5	42.7
GCLI-B (our)		64.0	45.7	43.2	27.0	42.7
-	YOLOv8	71.5	50.4	49.1	30.6	43.6
ECA		72.9	48.4	48.6	30.6	43.6
CBAM		74.1	46.1	48.9	30.4	43.7
simAM		69.7	50.4	49.5	30.9	43.6
CA		70.5	50.7	51.8	32.4	43.6
GC		72.3	49.0	48.6	30.7	43.7
GCLI-A (our)		71.0	50.5	49.5	30.7	43.6
GCLI-B (our)		72.1	50.5	52.1	32.5	43.6

## Data Availability

All datasets used in the study are open access, including th Chaoyang dataset at https://bupt-ai-cz.github.io/HSA-NRL/ (accessed on 21 May 2024), VinDR-CXR dataset at https://physionet.org/content/vindr-cxr/1.0.0/ (accessed on 21 May 2024), and GRAZPEDWRI-DX dataset at https://figshare.com/articles/dataset/GRAZPEDWRI-DX/14825193 (accessed on 21 May 2024).

## Abbreviations

The following abbreviations are used in this manuscript:

AP	average precision
mAP	mean average precision
avg	average
YOLOv8	You Only Look Once Version 8
RT-DETR	Real-Time DEtection TRansformer
CA	Coordinate Attention
ECA	Efficient Channel Attention
CBAM	Convolutional Block Attention Module
simAM	simple Attention Module
SE	Squeeze and Excitation
GC	Global Context
IoU	Intersection over Union
DL	deep learning
CNN	convolutional neural network
SOTA	state of the art
GCLI	Global Context and Local Interaction
SGD	Stochastic Gradient Descent
